# Protective effects of guarana (*Paullinia cupana*) against methotrexate‐induced intestinal damage in mice

**DOI:** 10.1002/fsn3.2101

**Published:** 2021-05-19

**Authors:** Adil Aldhahrani

**Affiliations:** ^1^ Department of clinical laboratory sciences Turabah University College Taif University Taif Saudi Arabia

**Keywords:** Bcl2, caspase 9, enteritis, IL‐1β, methotrexate, *Paullinia cupana*

## Abstract

This study aimed to examine the effects of guarana (*Paullinia cupana*) on intestinal damage induced by MTX in mice. Mice were classified into four groups: control, MTX, guarana (*Paullinia cupana*), and guarana (*Paullinia cupana*) together with MTX. Total antioxidant capacity together with glutathione, superoxide dismutase, MDA, ALT, AST, myeloperoxidase, total protein and IL‐1β were detected in the serum. Bax and Bcl2 expressions were detected in intestine together with histopathological examination and immunohistochemical examination of caspase‐9. Intoxication with MTX inhibited antioxidant and promoted myeloperoxidase activity in experimental mouse models but pre‐administration of guarana ameliorated this effect by inhibiting IL‐1β. Real‐time quantitative PCR (qRT‐PCR) analysis found that MTX intoxication upregulated BAX expression, causing apoptosis, and downregulated Bcl2 expression. These were also brought under control following guarana pre‐administration. Histological examination of intestine indicated hyperplasia and desquamation of superficial epithelium of villi in the MTX‐administered group, as well as round cell infiltration in the lamina propria. Pre‐administration of guarana protected against these effects. The MTX group showed that caspase‐9 expression was upregulated, increasing immune‐reactivity in comparison to the guarana experimental groups. These combined effects lead to the conclusion that guarana has a preventative or protective effect against MTX‐induced oxidative stress in the intestinal tissue.

## Graphical abstract

This study aimed to examine the effects of guarana (Paullinia cupana) on intestinal damage induced by MTX in mice. Total antioxidant capacity together with glutathione, superoxide dismutase, MDA, ALT, AST, myeloperoxidase, total protein, and IL‐1b were detected in the serum. Bax and Bcl2 expressions were detected in intestine together with histopathological examination and immunohistochemical examination of caspase‐9.

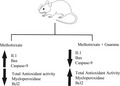

## 1 INTRODUCTION

Guarana (*Paullinia cupana*) is a natural herb, whose fruits contain high levels of caffeine and have been used more recently in energy and ‘sports’ branded drinks (Smith & Atroch, 2010). Guarana seed powder is used globally in nontraditional medicine (Bittencourt Lda et al., 2014) to combat mental fatigue and weight loss (de Oliveira Campos et al., 2011); as a ‘tonic’ for the heart, kidneys, and liver; to treat high cholesterol; to regulate digestion; and as an antiaging compound (Schimpl et al., 2013). Daily intake of guarana extract by elderly Amazonian humans has been linked with improved metabolism and reduced obesity (Krewer Cda et al., 2011). Research on guarana's biological effects does exist but information is scant on its effects on the intestine, particularly in relation to oxidative stress and enteritis caused by MTX. In light of guarana's wider benefits and increasing popularity, this study aims to explore the beneficial effects of guarana on the intestine following MTX intoxication in mice. MTX is a common chemotherapeutic agent and folic acid antagonist (Brown et al., 2016; Chan & Cronstein, 2013), which is also highly toxic. MTX induces oxidative stress in the heart, kidneys, and liver (Abdel‐Daim et al., 2017; Oktar et al., 2010; Oktem et al., 2006), but there has been no conclusive investigation of its effect on the intestine. Due to its effectiveness and widespread use in cancer treatment, understanding the side effects of MTX is of critical importance. Given the anecdotal and recorded therapeutic effects of guarana, investigating its ameliorative effects on MTX‐induced intestinal oxidative stress is a worthwhile pursuit.

## 2 MATERIALS AND METHODS

### 2.1 Materials

Aspartate transaminase (AST), alanine transaminase (ALT), malondialdehyde (MDA), reduced glutathione (GSH), superoxide dismutase (SOD), and total proteins were sourced from Biodiagnostic Company, Giza, Egypt. Reverse transcriptase, DNA ladder (100 bp), Oligo dT primers, SYBR Green PCR Master Mix, and TRIzol were from Invitrogen. The kits to determine total antioxidant activity (cat # TA 2513) were from Biodiagnostic Co. ELISA kits for mouse IL‐1β (ab197742) and myeloperoxidase (ab155458) were from Abcam Co.

### 2.2 Animals and experimental design

The College of Pharmacy, King AbdelAziz University, Saudi Arabia, provided 40 disease‐free 10‐week‐old, 35 g experimental male mice. These were manually handled for 10 days prior to the study to habituate them to human contact. Ethical approval for this study was granted the ethical committee at Turabah University. The mice were kept at room temperature (25 ± 5°C) in the laboratory house at Turabah University and given access to food and water and then divided into four groups of ten mice as follows:

Group 1: Control (CNT), given access to food and water only.

Group 2: MTX‐intoxicated group, injected intraperitoneally (20 mg/kg bw, single dose on day 7).

Group 3: Administered 300 mg/kg bw orally with *Paullinia cupana* seed extract for 12 days.

Group 4: Administered guarana as above for *seven* days. Administered MTX on day seven (as above) then continued to take guarana for a further five days (12 days total).

Following the experimental phase, the mice were anaesthetized with dimethyl ether then decapitated. Intestinal tissue was collected and preserved in 10% formalin for histopathological and immunohistochemical examination. Other part was flash frozen in liquid nitrogen for real‐time PCR. Blood was collected from medial canthus of the eye to obtain serum for biochemical analysis.

### 2.3 Biochemical and antioxidants assessment

Total antioxidant capacity (TAC) in serum, myeloperoxidase and serum ALT and AST levels were measured using a colorimetric spectrophotometer based on the manufacturer's instructions. ELISA kits were used to measure IL‐1β according to the manufacturer's instructions. Serum MDA was assessed according to the method defined by (Ohkawa et al., 1979), while GSH and SOD were measured using the methods of (Beutler et al., 1963; Nishikimi et al., 1972), and total proteins per (Lowry et al., 1951).

### 2.4 Quantitative real‐time PCR (qRT‐PCR)

RNA was extracted from samples of intestinal tissue using the method described by Soliman et al. (Soliman et al., 2020). Samples were frozen then homogenized before adding chloroform and centrifuging at 4°C. Isopropanol was added in equal measure to the supernatant. RNA pellets were separated out and washed in 70% alcohol and then dissolved in DEPC‐treated H_2_O. After being incubated in a T100TM Thermal Cycler (Bio‐Rad) for five minutes at 70°C, denaturation of 3 µg of extracted RNA together with 0.5 ng oligo dT (Invitrogen) occurred. 2 µl of 10 mM dNTPs, 100 U of M‐MuLV (Qiagen) and 10× RT‐buffer (2 µl) was added to reverse transcribe the denatured RNA. This was then incubated again at 37°C for one hour and then at 90°C for ten minutes to inactivate any remaining enzymes. qRT‐PCR primers were designed using GenScript Real‐time PCR (TaqMan) Primer Design and these are detailed in Table 1.

**TABLE 1:** Real‐time PCR primers used in this study

	Gene	Primer sequence	Accession number	Product size (bp)	Annealing temp (°C)
BAX	BAX‐F	CGCGTGGTTGCCCTCTTCTA	NM_007527	153	60
	GLP−2‐R	TTCCCAGCCACCCTGGTCTT			
Bcl2	Bcl2‐F	AGCCTGAGAGCAACCCAAT	NM_009741	159	60
	Bcl2‐R	AGCGACGAGAGAAGTCATCC			
β‐actin	β‐actin‐F	CCAGCCTTCCTTCTTGGGTA	NM_007393.5	143	60
	β‐actin‐R	CAATGCCTGGGTACATGGTG			

PCRs comprised of f 1 μg/μl cDNA (1.5 μl), SYBR Green PCR Master Mix (10 μl). To this was added 1 μM of forward and reverse primer for each gene to be examined and the mixture was made up to 20 μl with nuclease free H_2_O. Applied Biosystems 7500 Fast Real‐Time PCR Detection system was run using the following conditions: 94°C for ten minutes (first denaturation); forty cycles of 94°C for 20 s (second denaturation); 60°C for one minute (annealing and extension). The critical threshold (Ct) of the target gene was normalized using quantities (Ct) of the housekeeping gene (β‐actin), using the formula x = 2−ΔΔCt, where there is x = fold difference relative to the control.

### 2.5 Histopathological examination

Intestinal tissue samples were obtained from the duodenum, preserved in 10% formalin for 24 hr, washed, mixed with ethanol at varying concentrations then cleared using xylene, embedded in soft and hard paraffin, then sectioned and stained using hematoxyline and eosin (H&E).

### 2.6 Immunohistochemical examination of caspase‐9

Tissue sections were deparaffinized using xylene and 3% H_2_O_2_, inactivating the peroxidases. Antigen retrieval was carried out using citrate buffer (10 mM) for 30 min at 121°C. 5% normal serum was added as a blocking agent, to which was added a rabbit polyclonal anti‐caspase‐9 antibody (1:100; sc‐53566; Santa Cruz Biotechnology, Inc.), then incubated at 4°C overnight. Samples were washed thoroughly in PBS then incubated with a goat anti‐rabbit IgG–biotin‐conjugated secondary antibody (1:2,000; sc‐2040; Santa Cruz Biotechnology, Inc.) for 20 min at 32°C. Horseradish peroxidase‐labeled streptavidin was added to the mixture and diaminobenzidine (DAB) was used to visualize antibody binding. Tissue sections were then counterstained using hematoxylin. Immunohistochemical scoring was detected as follow: Score 1 = (weak; < 10 positive stained cells (caspase‐9) per each of three high‐power fields (HPF), at 40 × magnification; Score 2 = (moderate; 11–20 positive stained cells/HPF) Score 3 = (strong; > 20 positive stained cells/HPF).

### 2.7 Statistical analysis

Results are reported as the means for seven mice from each group ± standard error of means (*SEM*). Data were inputted into SPSS software (IBM), which carried out one‐way ANOVA test. *p* < .05 was considered statistically significant.

## 3 RESULTS

### 3.1 Effects of guarana on liver enzymes

AST and ALT levels were raised in the liver tissues of MTX‐intoxicated mice, while total protein levels decreased. These changes were less pronounced in the guarana pretreated mice (Table 2).

**TABLE 2:** Effects of guarana on mouse liver biomarkers following MTX intoxication

Parameter group	ALT (U/l)	AST (U/l)	Total proteins (mg/dl)
Control	16.1 ± 1.1	20.1 ± 1.9	71.1 ± 4.8
Guarana	20.5 ± 2.0	19.3 ± 1.3^a^	74.4 ± 1.9
Methotrexate	87.3 ± 3.1^a^	77.5 ± 7.2^a^	39.8 ± 3.8^a^
Guarana + methotrexate	36.6 ± 2.7^b^	30.6 ± 3.6^b^	73.2 ± 3.9^b^

Note

Abbreviations: ALT, alanine transaminase; AST, aspartate transaminase.

Values are means ± standard error (*SEM*) for 7 mice per group.

Values are statistically significant at ^a^p < 0.05 versus. control and guarana groups. ^b^p < 0.05 versus. methotrexate

### 3.2 Effects of guarana on total antioxidant capacity and myeloperoxidase levels

Total antioxidant capacity (TAC) decreased in MTX‐administered mice (Table 3), whereas the guarana‐only administered group showed healthy levels of antioxidant activity. For the MTX group pre‐administered with guarana, TAC levels showed no change after MTX intoxication. Myeloperoxidase (MPO) decreased marginally following MTX intoxication but the effect was also ameliorated by guarana (Table 2). Table 3 shows the effects of guarana pretreatment on other antioxidants. Increased serum levels of MDA, along with decreased SOD and GSH indicates tissue degradation. While guarana increased SOD and GSH levels, this was counterbalanced by MTX intoxication.

**TABLE 3:** Effects of guarana on antioxidant activity in MTX‐intoxicated mice

Parameter group	TAA (mM/ml)	MPO (ng/ml)	MDA (nmol/ml)	SOD (U/ml)	GSH (nmol/l)
Control	70 ± 7	242 ± 17.7	21.9 ± 0.3	5 ± 0.2	3.6 ± 0.3
Guarana	98 ± 14	248 ± 7	20.3 ± 0.2	6.1 ± 0.5	4.2 ± 0.4
Methotrexate	45 ± 11	208 ± 14	62.2 ± 4.5^a^	2.4 ± 0.4^a^	1.3 ± 0.03^a^
Guarana + MTX	69 ± 12	220 ± 13	35.8 ± 2.4^b^	4.1 ± 0.6^b^	2.4 ± 0.3^b^

Note

Abbreviations: GSH, glutathione; MDA, malondialdehyde; MPO, myeloperoxidase; SOD, superoxide dismutase; TAA, total antioxidant activity.

Values are means ± *SEM* for 7 mice per experiment.

Values are statistically significant at ^a^p < 0.05 versus. control and guarana groups; ^b^p < 0.05 versus. MTX.

### 3.3 Effects of guarana on serum interleukin‐1β levels altered by methotrexate

**FIGURE 1:**
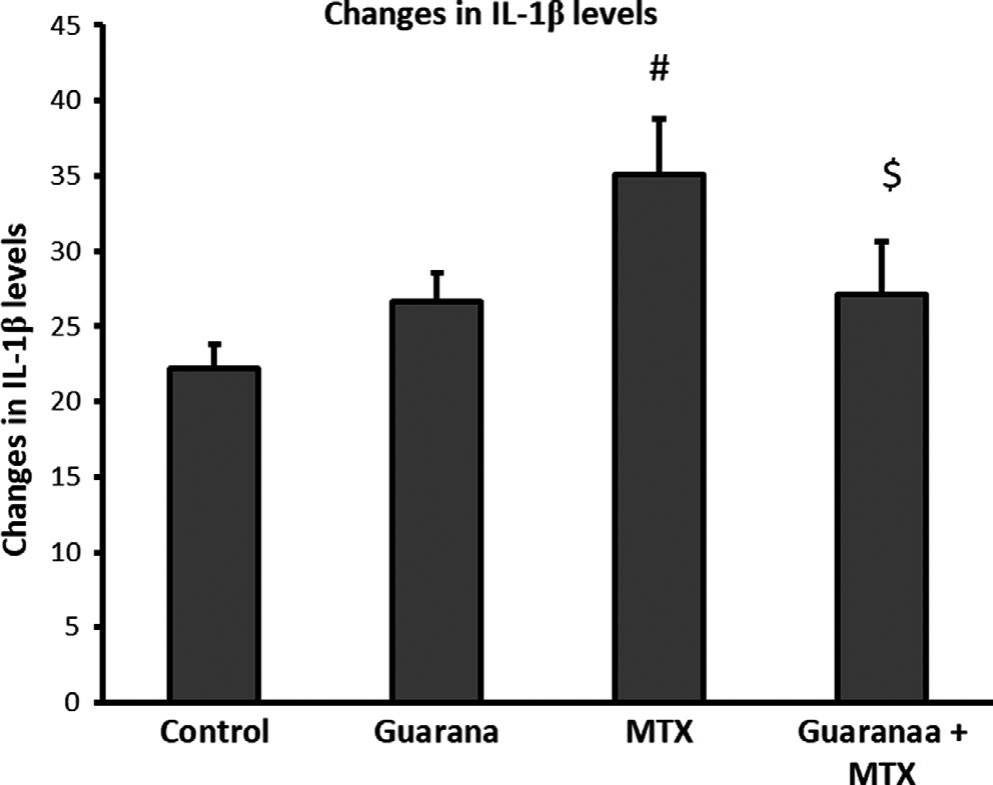
Ameliorative effects of guarana on serum IL‐1β levels (pg/ml) in MTX‐administered mouse. Values are means ± *SEM* for 7 different mice per each experiment. Values are statistically significant at *p* < .05 versus. control (*) and guarana (^#^) groups and $*p* < .05 versus. MTX group. MTX; methotrexate

MTX intoxication increased levels of IL‐1β, a pro‐inflammatory cytokine (Figure 1) but guarana pre‐administration protected against this effect (Figure 1).

### 3.4 Apoptotic and anti‐apoptotic gene expression in methotrexate‐intoxicated mice

**FIGURE 2:**
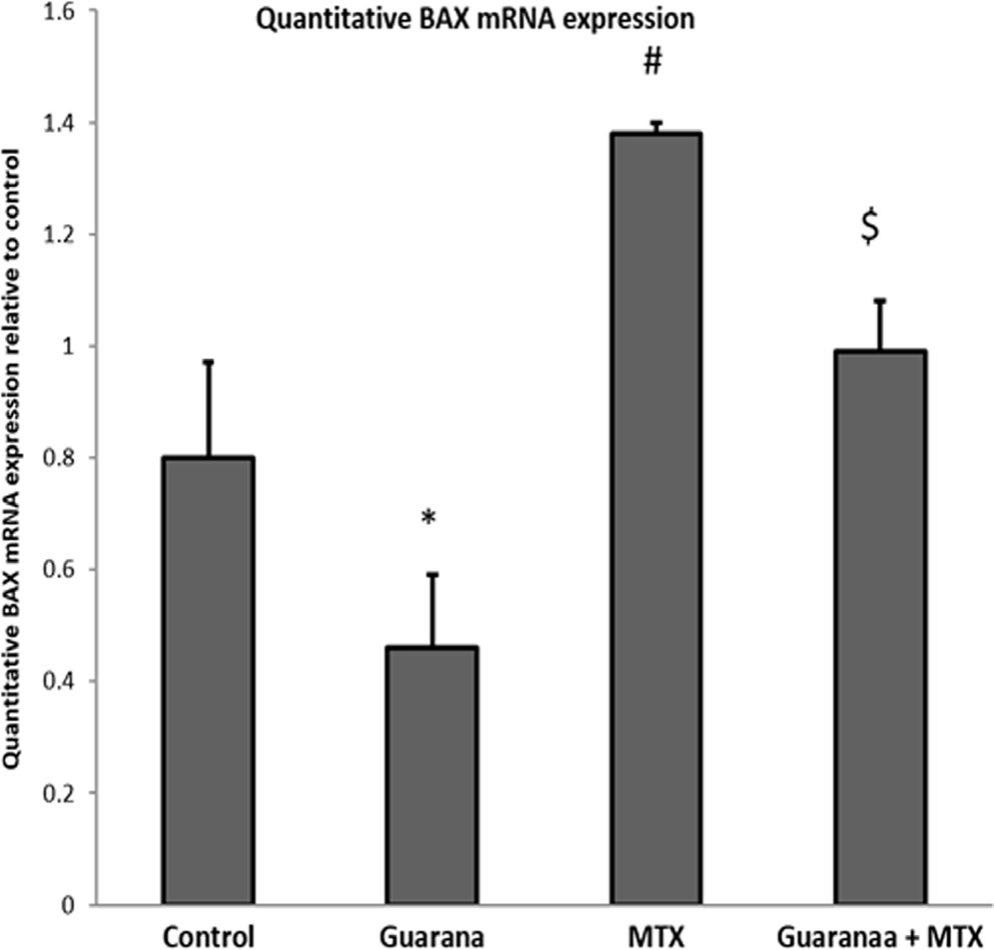
Ameliorative impacts of guarana on mRNA expression BAX using quantitative real‐time PCR. Graphic presentation of intestinal mRNA BAX in different groups of mice after normalization with beta actin. Values are statistically significant at *p* < .05 versus. control (*) and guarana (#) groups and $*p* < .05 versus. MTX group. MTX, methotrexate

**FIGURE 3:**
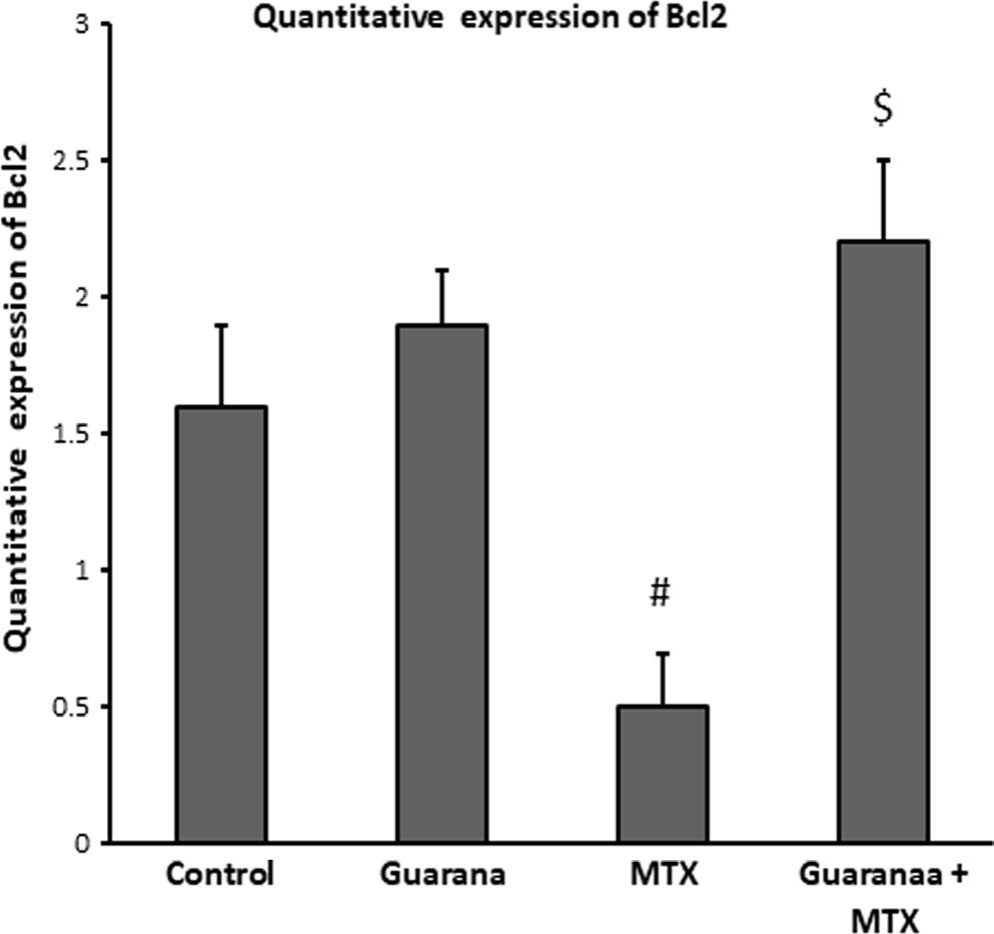
Ameliorative impacts of guarana on mRNA expression Bcl2 using quantitative real‐time PCR. Graphic presentation of intestinal mRNA Bcl2 in different groups of mice after normalization with beta actin. Values are statistically significant at *p* < .05 Vs control (*) and guarana (#) groups and $*p* < .05 versus. MTX group. MTX: methotrexate

MTX caused an increase in BAX expression (Figure 2), which regulates apoptosis in the intestine, and downregulated mRNA expression of Bcl2, an anti‐apoptotic gene (Figure 3). Mice pre‐administered with guarana showed downregulated of BAX mRNA and normal expression of Bcl2 mRNA.

### 3.5 Results of histopathological examination

**FIGURE 4:**
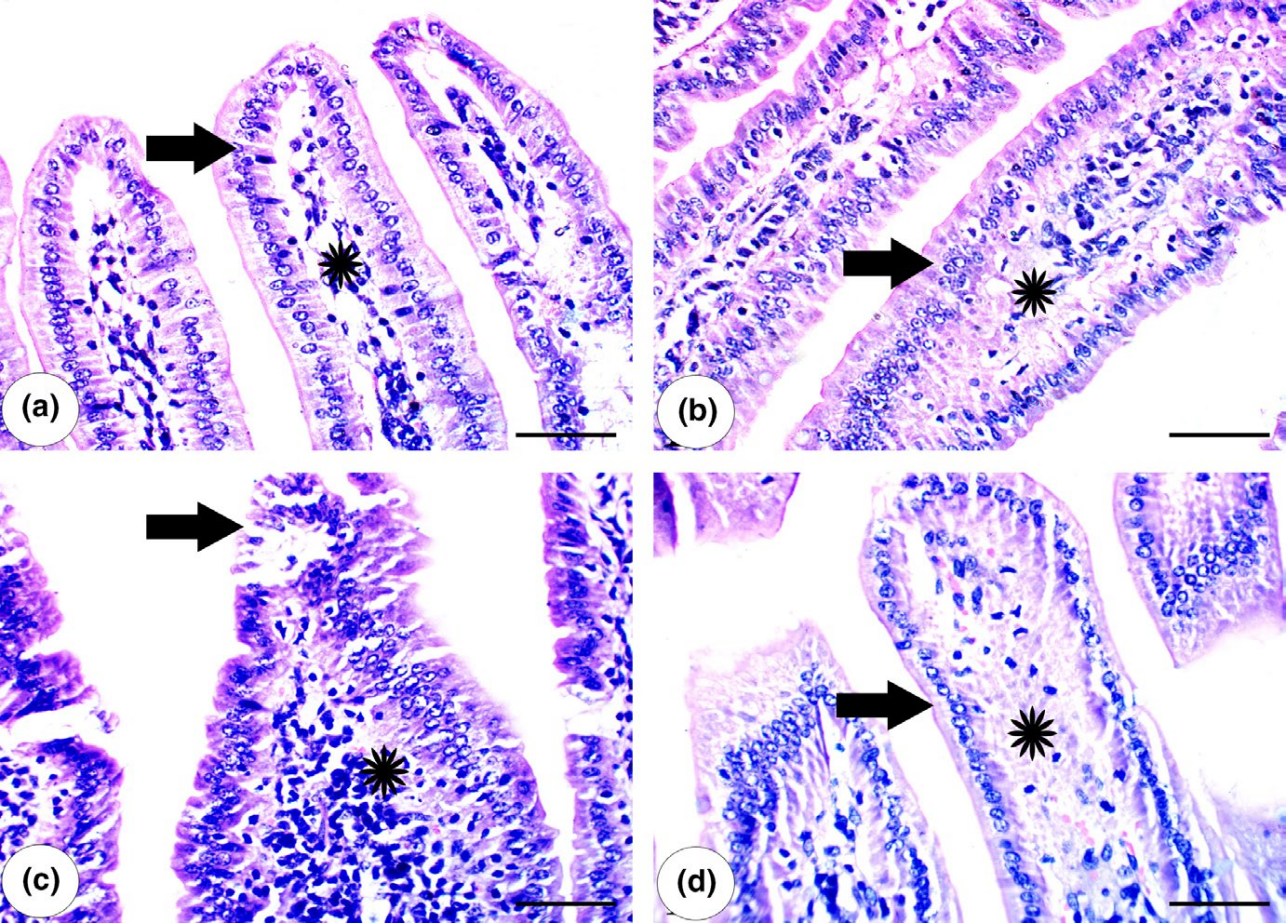
Intestine Histology: (a) Intestine of control mouse showed normal villi with normal tissue architecture of lamina epithelialis (arrow) and lamina propria (*). (b) Intestine of guarana‐administered group showed normal columnar epithelium (arrow) and normal lamina propria (*). (c) Intestine of methotrexate‐administered group showing hyperplasia and desquamation of superficial epithelium of villi (arrow) with extensive infiltration of round cells in the lamina propria (*). (d) Intestine of methotrexate‐administered group treated with guarana showing regeneration of lamina epithelialis (arrow) and lamina propria (*). Scale bar = 50 µm

Intestinal samples from the CNT group exhibited normal villi as well as normal tissue architecture in both the lamina propria and lamina epithelialis (Figure 4a). The guarana‐only group exhibited normal lamina propria and columnar epithelium (Figure 4b). The MTX‐administered group exhibited desquamation of the villous superficial epithelium, extensive round cell infiltration in the lamina propria, and hyperplasia (Figure 4c). The guarana pre‐administered group exhibited normal lamina propria and columnar epithelial architecture (Figure 4d).

### 3.6 Immunohistochemical examination of caspase‐9

**FIGURE 5:**
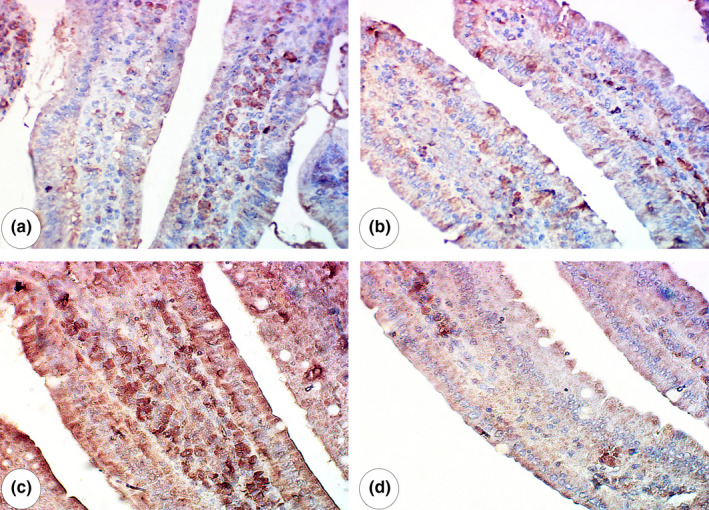
Immunohistochemical examination of caspase‐9 (a) & (b) Intestine of control and guarana‐administered groups respectively showed mild expression of caspase‐9. (c) Intestine of methotrexate‐administered group showed overexpression of caspase‐9 in the villus mucosa. (d) Intestine of methotrexate‐administered group treated with guarana showed moderate caspase‐9 expression in the villous mucosa. Scale bar = 50 µm

The CNT and guarana‐only groups exhibited mild caspase‐9 expression in the intestinal villous mucosa (Figure 5a,b). The MTX group showed significantly increased caspase‐9 expression (Figure 5c), while the guarana pre‐administered group exhibited moderate caspase‐9 expression (Figure 5d). Immunohistochemical scoring showed strong expression of caspase‐9 in MTX group in relation to control group with weak expression in case of protection with guarana as seen in Table 4.

**TABLE 4:** Immunohistochemical scoring of caspase‐9 in intestinal sections of different groups

Immunohistochemical staining	C	G	MTX	G + MTX
Insulin	1	1	3	1

Note

C (control), G (guarana), MTX (methotrexate).

**FIGURE 6:**
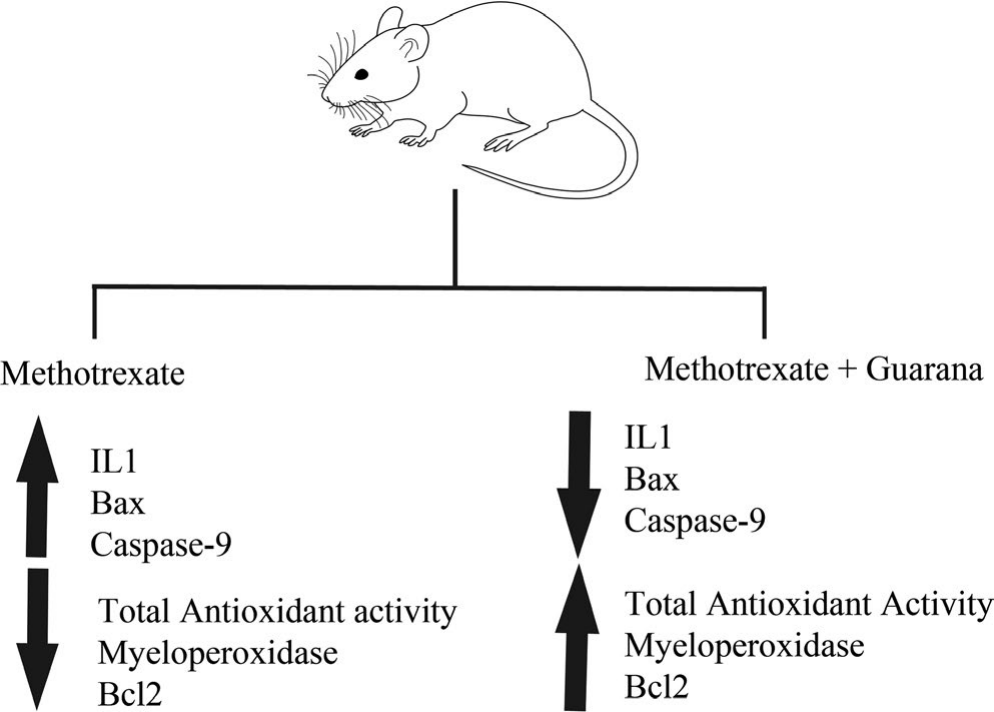
Summary figure Summary of Ameliorative effects of guarana on total antioxidant activity, myeloperoxidase, interleukin‐1 levels, Bax, Bcl2, and caspase expression

The results of total antioxidant activity, myeloperoxidase, interleukin‐1 levels, Bax, Bcl2, and caspase‐9 expressions are summarized in Figure 6.

## 4 DISCUSSION

AST and ALT in serum as well as blood urea nitrogen (BUN) levels were increased significantly by MTX intoxication, while antioxidants were decreased, resulting in higher levels of oxidative stress overall. Pretreatment with guarana undid and ameliorated this effect, thereby reducing oxidative stress and exhibiting anti‐oxidative and anti‐apoptotic functions.

A number of antitumor molecules have been developed using natural organisms, including marine animal, plants, and microorganisms, from which more than 60% of cancer therapy drugs are derived (Cragg & Newman, 2005). Medicinal plants have been used as traditional treatments for several diseases for many decades and in different parts of the world. In rural parts of developing countries, they remain the primary source of medicine (Kumar et al., 2019). Some plants can also be used to ameliorate the toxic side effects of chemotherapeutic agents including MTX, although this is less well researched. This study explored the usage of guarana in protecting against MTX‐induced oxidative stress in the intestine and in helping to control apoptosis/anti‐apoptosis. Several studies recommended that MTX is the most applicable chemotherapeutic agent with numerous symptoms of toxicity (Widemann & Adamson, 2006; Widemann et al., 2004). Moreover, it may result in second malignancy, which is associated with many therapeutic treatments (Guidi et al., 2018). Methotrexate is used in chemotherapy of various malignancies and as a treatment for inflammatory conditions. One of the common adverse effects of MTX treatment is intestinal inflammation due to increase of the oxidant parameters and decrease of the antioxidant parameters (Ozcicek et al., 2020). In this study, MTX significantly decreased total antioxidant activity. This oxidative stress was protected by pre‐administration of guarana seed extract as reported by (Majhenič et al., 2007). The toxic effects of MTX and other chemotherapy drugs are due to oxidative stress and the formation of reactive oxygen species (Widemann & Adamson, 2006). As reported, methotrexate stimulated production of free radicals from both endogenous and exogenous sources (Famurewa et al., 2017). Literature proved oxidative stress implication in MTX toxicity (Famurewa et al., 2017). Oxidative stress induced by MTX‐induced depletion in total antioxidant and MPO activities, along with reduced SOD and GSH (Sheikh et al., 2014). Myeloperoxidase is a marker of inflammatory response are increased and antioxidants activity was decreased in MTX‐induced intestinal inflammation (Ozcicek et al., 2020). SOD and GSH are key constituents within antioxidant defense systems. All these side effects were protected and remained within normal ranges by pre‐administration of guarana (Tables 2, 3). AST and ALT were increased following MTX intoxication, lowering total proteins, and damaging liver tissues. Pretreatment with guarana ameliorated all of these effects.

Previous studies (Famurewa et al., 2019; Sheikh et al., 2014) confirmed that toxicity of methotrexate is associated with elevated inflammatory cytokine production. In this study, methotrexate increased the production of IL‐1β. Administration of guarana alone or prior to MTX prevented the harmful effects induced by MTX. Guarana improved the anti‐inflammatory state by upregulation of Il‐1β secretion. Antioxidant activity and inhibition of IL‐1β may help in the prevention of MTX intestinal inflammation (Ozcicek et al., 2020). IL‐1β is one of the most potent mediators of inflammation, which induce inflammatory reaction via binding to IL‐1β receptor 1 (Yazdi & Ghoreschi, 2016). Moreover, da Costa Krewer et al., (2014) in vivo and in vitro studies proved effective anti‐inflammatory effect of guarana by decreasing IL‐1β level in mice model (da Costa Krewer et al., 2014). Robust evidence suggests that guarana is widely used as a stimulant. Its seeds can contain up to 6% caffeine (Schimpl et al., 2013), although a range of other biological effects have been observed, including antioxidant activity (Basile et al., 2005; Mattei et al., 1998), antimicrobial effects (Yamaguti‐Sasaki et al., 2007), anticarcinogenic properties (Leite et al., 2011), anti‐depressive effect (Otobone et al., 2007), and the potential to help with weight loss (Opala et al., 2006). Caffeine has shown potential anticarcinogenic effects that may be related to its antioxidant and antimutagenic effects. Also, caffeine may specifically protect against distal colon cancer by increasing the motility of the distal colon and, as a consequence, lowering carcinogen exposure of colonic epithelial cells (Dik et al., 2014).

Our study found guarana's antioxidant properties to be effective in counteracting the reduced intestinal antioxidant potential and decreased TAC and MPO levels caused by MTX intoxication. Oxidative stress usually originates from imbalance of prooxidant–antioxidant, causing cellular damage as seen with MTX treatment. Its action is related mainly to free radicals, such as reactive oxygen or nitrogen species, that are produced during the course of pathological inflammatory processes (Baek & Lee, 2016). Tissue MPO activity is one of the earliest consequences of inflammation that could be used for evaluation of the oxidative stress level (Tóth et al., 2017). Decreased MPO activity as seen in MTX group results in the exaggeration of inflammatory response (Aratani, 2018).

Guarana's antitumor properties have been observed in animal models and cellular experimental studies (Fukumasu et al., 2006; Ifergan et al., 2004). Furthermore, across all the chemotherapeutic drugs that were studied, it was found that 72 hr of guarana exposure improves anti‐proliferative activity and therefore does not compromise chemotherapeutic activity. For this reason, and due to its stimulant properties, guarana is considered a valuable treatment for chemotherapy‐induced cancer‐related fatigue (CRF).

Cells’ susceptibility to degradation, destruction, and apoptosis is controlled by the genes for Bax, a pro‐apoptotic protein, and Bcl‐2, which regulates apoptosis (Almeida et al., 2000). Inflammatory cytokines and oxidative stress activate Bax (Mahmoud et al., 2019), whereas caspases activation causes inhibition of Bcl‐2 (Yang et al., 1997). Bax and Bcl‐2 genes belong to Bcl‐2 family, that regulates apoptosis (Almeida et al., 2000). Bax is a pro‐apoptotic gene activated by oxidative stress (Mahmoud et al., 2019). Unlike Bax, Bcl‐2 blocks and antagonizes programmed cell death (apoptosis) (Yang et al., 1997). MTX intoxication causes caspase‐9 and Bax to be upregulated which indicates incidence of apoptosis and Bcl‐2 expression as anti‐apoptotic to be downregulated (Figures 4, 5). Pre‐administration with guarana ameliorated these effects in mice, preventing cell death by suppressing expression of Bax, and caspase‐9 expressions, and activating Bcl‐2 expression, which inhibits apoptosis.

## 5 CONCLUSION

This study found that guarana (*Paullinia cupana*) protected against MTX‐induced intestinal tissue oxidative stress. This was facilitated through increased MPO and antioxidant activity, and decreased IL‐1β. Bax and caspase‐9 expression was inhibited, while Bcl‐2 was activated, indicating guarana's anti‐apoptotic properties.

## CONFLICT OF INTEREST

The author declares that no conflicts of interest exist.

## Data Availability

The materials and data included within the study are available from the corresponding author upon reasonable request.
